# With a Little Help from a Friend

**DOI:** 10.1371/journal.pbio.0050190

**Published:** 2007-07-17

**Authors:** Frans B. M de Waal

## Abstract

In humans, the most commonly assumed motivation behind altruism is empathy. Might this also apply to other animals or are they indifferent to each other's welfare?

An old female, named Peony, spends her days with other chimpanzees in a large outdoor enclosure near Atlanta, Georgia (United States). On bad days, when her arthritis is acting up, she has great trouble walking and climbing. But other females help her out. For example, Peony is huffing and puffing to get up into the climbing frame in which several chimpanzees have gathered for a grooming session. An unrelated younger female moves behind her, places both hands on her ample behind, and pushes her up with quite a bit of effort, until Peony joins the rest.

I collect cases like this and have hundreds of them, because the issue of altruism remains profoundly interesting to the biologist (Figure 1). Why do animals care about each other: should not they just care about themselves? Modern textbooks offer the impression that the altruism question has been resolved, but this applies only to one half of it—the part about evolutionary origin. Altruistic tendencies are thought to have evolved to help either kin or those willing and capable of returning the favor. Helping of kin can be explained by shared genes: instead of advancing one's own genes, one helps other carriers of the same genes [1]. The second kind of altruism rewards the performer with return-benefits— “I'll scratch your back, if you scratch mine” —resulting in a net gain for both parties [2]. In this view, altruistic behavior ultimately benefits either the organism itself or its immediate kin, which explains Robert Trivers' claim that “models that attempt to explain altruistic behavior in terms of natural selection are models designed to take the altruism out of altruism” [2 (p. 35)].

**Figure 1:**
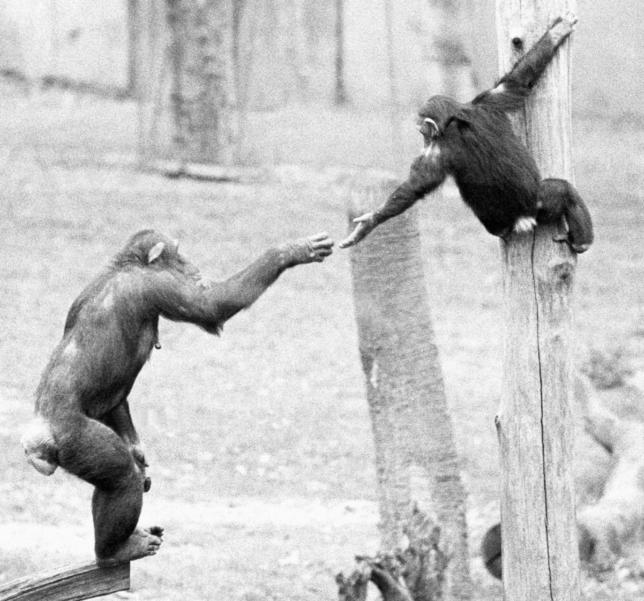
Apes Often Show So-Called “Targeted Helping,” Which Is Aid Tailored to the Other's Specific Needs In this case, a mother chimpanzee reaches out to help her juvenile son out of a tree after he screamed and begged (see hand gesture). (Photograph by F.B.M. de Waal)

The above is often translated by saying that animals “cooperate for selfish reasons,” but this statement is problematic. Perhaps we know the evolutionary reasons for their behavior, but does this really amount to knowing the animals' reasons? How does an animal arrive at its decision to help another? This is the other half of the question. Here, the issue is not past natural selection, but the current situation. Clearly, return-benefits matter only insofar as they are knowable to the actors. Most of the time, though, the benefits of altruistic behavior occur so distantly in time—if they occur at all—that it will be hard or impossible for animals to connect them with the original act.

Once evolved, behavior often assumes motivational autonomy: its motivation becomes disconnected from evolutionary goals. A good example is sexual behavior, which arose to serve reproduction. Since animals are, so far as we know, unaware of the link between sex and reproduction, they must be engaging in sex (as do humans much of the time) without progeny in mind. Just as sex cannot be motivated by unforeseen consequences, altruistic behavior cannot be motivated by unforeseen pay-offs.

The motivation to help must therefore be relatively autonomous and stem from immediate factors, such as a sensitivity to the needs of others. Such sensitivity would by no means contradict the self-serving reasons why altruism evolved, so long as it steers altruistic behavior in the direction predicted by theories of kin selection and reciprocal altruism [3].

In humans, the most commonly assumed motivation behind altruism is empathy. We identify with another in need, pain, or distress, which induces emotional arousal that may translate into sympathy and helping [4]. Inasmuch as there are signs of empathy in other animals, from rodents to primates, the same hypothesis may apply [3,5]. This can be tested by evaluating how animals perceive another's situation and under which circumstances they try to ameliorate this situation.

Apart from assisting an “old lady” in her climbing efforts, chimpanzees occasionally perform extremely costly actions. For example, when a female reacts to the screams of her closest associate by defending her against a dominant male, she takes enormous risks on behalf of the other. She may very well get injured. Note the following description of two long-time chimpanzee friends in a zoo colony: “Not only do they often act together against attackers, they also seek comfort and reassurance from each other. When one of them has been involved in a painful conflict, she goes to the other to be embraced. They then literally scream in each other's arms” [6 (p. 67)]. This kind of cooperation, expressed in alliances and coalitions, is among the best-documented in primatology [7].

Some of the costliest helping behavior occurs in relation to water: “In some zoos, chimpanzees are kept on man-made islands, surrounded by water-filled moats…Chimpanzees cannot swim and, unless they are rescued, will drown if they fall into deep water. Despite this, individuals have sometimes made heroic efforts to save companions from drowning—and were sometimes successful. One adult male lost his life as he tried to rescue a small infant whose incompetent mother had allowed it to fall into the water” [8 (p. 213)]. Explanations of such behavior on the basis of possible return-benefits make a huge cognitive leap by attributing long-term expectations to chimpanzees. More likely, such rescue attempts are emotionally driven, because hydrophobia can be overcome only by an overwhelming motivation.

This brings us to the study conducted at the Ngamba Island Chimpanzee Sanctuary, in Uganda, by Felix Warneken and colleagues [9] of the Max-Planck Institute for Evolutionary Anthropology, in Leipzig, Germany. The investigators set out to determine the precise circumstances under which chimpanzees are willing to assist either humans or each other. The investigators tried to rule out reciprocity by having the apes interact with humans they barely knew, and on whom they had never depended for food or other favors (see also the research article by Rutte and Taborsky in this issue of *PLoS Biology*, doi:10.1371/journal.pbio.0050196). They also tried to rule out the role of immediate return-benefits by manipulating the availability of rewards.

One important strength of this study is that three different experiments were conducted, all hinting in the same direction. In the first experiment, the chimpanzee saw a person unsuccessfully reach through the bars for a stick on the other side, too far away for the person, but within reach of the ape. The chimpanzees spontaneously helped the reaching person regardless of whether this yielded a reward, or not. A similar experiment with 18-month-old children gave exactly the same outcome. Obviously, both apes and young children are willing to help, especially when they see someone struggling to reach a goal.

The second experiment increased the cost of helping. The chimpanzees were still willing to help, however, even though now they had to climb up a couple of meters, and the children still helped even after obstacles had been put in their way. Rewards had been eliminated altogether this time, but this hardly seemed to matter.

One could, of course, argue that chimpanzees living in a sanctuary help humans because they depend on them for food and shelter. How familiar they are with the person in question may be secondary if they simply have learned to be nice to the bipedal species that takes care of them. The third and final experiment therefore tested the apes' willingness to help each other, which, from an evolutionary perspective, is also the only situation that matters.

The set-up was slightly more complex. One chimpanzee, the Observer, would watch another, its Partner, try to enter a closed room with food. The only way for the Partner to enter this room would be if a chain blocking the door were removed. This chain was beyond the Partner's control—only the Observer could untie it. Admittedly, the outcome of this particular experiment surprised even me—and I am probably the biggest believer in primate empathy and altruism. I would not have been sure what to predict given that all of the food would go to the Partner, thus creating potential envy in the Observer. Yet, the results were unequivocal: Observers removed the peg holding the chain, thus yielding their Partner access to the room with food.

To create the right conditions experimentally for helping behavior is less obvious than it may appear, as illustrated by two recent failures to find altruism in chimpanzees [10,11]. In these studies, apes ignored the good of others while pursuing immediate gains for themselves. Interpreted as proof of unmitigated selfishness, the results of these studies were played up in one title as “chimpanzees are indifferent to the welfare of unrelated group members” [10]. But all that these experiments really showed was that humans can create situations in which apes focus on their own interests. With regards to our own species, too, it will not be hard to create such situations. Take the way people often trample each other to get to the merchandise as soon as a department store opens its doors for a major sale. Would anyone conclude from these scenes that humans, as a species, are indifferent to each other's welfare?

It is well known that absence of evidence is not evidence of absence. The reason this mantra of experimental psychology was ignored in relation to earlier negative findings may relate to an influential school of thought according to which human altruism is absolutely unique [12–14]. The earlier findings seemed to support this view. This school is running into increasing trouble, however, now that we know that apes often ignore kinship when cooperating [15], show remarkable levels of empathy and spontaneous assistance [16,17], and in experimental settings, such as discussed here, assist both humans and conspecifics in a seemingly disinterested manner. The difference with human behavior may be smaller than assumed.

This is, of course. the refrain of all ape research over the past couple of decades, and warns against the assumption of discontinuity that remains popular outside of biology. The study by Warneken et al. [9] further adds an interesting element that begs further exploration: immediate reward is apparently irrelevant for the observed behavior. One would think that rewards, even if not strictly necessary, would at least stimulate helping behavior, but in fact they seem to play no role at all. This seems to suggest that the decision to help is not based on a cost/benefit calculation, as is so often assumed. The responses observed in these experiments seem genuinely other-oriented.

Perhaps it is time to abandon the idea that individuals faced with others in need decide whether to help, or not, by evaluating costs and benefits on the spot. Instead, natural selection may have made these calculations for them. Weighing the consequences of behavior over evolutionary time, natural selection has produced psychological mechanisms designed to produce spontaneous helping that, on average and in the long run, works to the advantage of both actors and recipients. To put this in biological language: natural selection has produced a proximate mechanism that takes care of ultimate goals [3]. This is, of course, how all behavioral evolution proceeds. After a period with a narrow focus on evolutionary reasons for altruistic behavior, we should start paying more attention to how exactly such behavior is produced in the here and now.

## References

[pbio-0050190-b001] Hamilton WD (1964). The genetical evolution of social behaviour I and II. J Theor Biol.

[pbio-0050190-b002] Trivers RL (1971). The evolution of reciprocal altruism. Quant Rev Biol.

[pbio-0050190-b003] de Waal FBM (2007). Putting the altruism back into altruism: The evolution of empathy. Annu Rev Psychol.

[pbio-0050190-b004] Batson CD (1991). The altruism question: Toward a social-psychological answer.

[pbio-0050190-b005] Preston SD, de Waal FBM (2002). Empathy: Its ultimate and proximate bases. Behav Brain Sci.

[pbio-0050190-b006] de Waal FBM (1998 [1982]). Chimpanzee politics: Power and sex among apes.

[pbio-0050190-b007] Harcourt AH, de Waal FBM (1992). Coalitions and alliances in humans and other animals.

[pbio-0050190-b008] Goodall J (1990). Through a window: My thirty years with the chimpanzees of fombe.

[pbio-0050190-b009] Warneken F, Hare B, Melis AP, Hanus D, Tomasello M (2007). Spontaneous altruism by chimpanzees and young children. PLoS Biol.

[pbio-0050190-b010] Silk JB, Brosnan SF, Vonk J, Henrich J, Povinelli D (2005). Chimpanzees are indifferent to the welfare of unrelated group members. Nature.

[pbio-0050190-b011] Jensen K, Hare B, Call J, Tomasello M (2006). What's in it for me? Self-regard precludes altruism and spite in chimpanzees. Proc R Soc London Ser B.

[pbio-0050190-b012] Dawkins R (1976). The selfish gene.

[pbio-0050190-b013] Fehr E, Fischbacher U (2003). The nature of human altruism. Nature.

[pbio-0050190-b014] Richerson PJ, Boyd R (2005). Not by genes alone.

[pbio-0050190-b015] Langergraber KE, Mitani JC, Vigilant L (2007). The limited impact of kinship on cooperation in wild chimpanzees. Proc Nalt Acad Sci U S A.

[pbio-0050190-b016] de Waal FBM (1996). Good natured: The origins of right and wrong in humans and other animals.

[pbio-0050190-b017] de Waal FBM (1997). Bonobo: The forgotten ape.

